# Improvements of ^177^Lu SPECT images from sparsely acquired projections by reconstruction with deep-learning-generated synthetic projections

**DOI:** 10.1186/s40658-024-00655-x

**Published:** 2024-06-28

**Authors:** Emma Wikberg, Martijn van Essen, Tobias Rydén, Johanna Svensson, Peter Gjertsson, Peter Bernhardt

**Affiliations:** 1 Department of Medical Radiation Sciences, Institute of Clinical Sciences, Sahlgrenska Academy, University of Gothenburg, Gothenburg, Sweden; 2 Department of Molecular and Clinical Medicine, Institute of Medicine, Sahlgrenska Academy, University of Gothenburg, Gothenburg, Sweden; 3 Department of Clinical Physiology, Sahlgrenska University Hospital, Gothenburg, Sweden; 4 Medical Physics and Medical Bioengineering, Sahlgrenska University Hospital, Gothenburg, Sweden; 5 Department of Oncology, Institute of Clinical Sciences, Sahlgrenska Academy, University of Gothenburg, Gothenburg, Sweden

**Keywords:** SPECT imaging, SPECT reconstruction, Dosimetry, AI, Deep-learning, Molecular radiotherapy, Radionuclide therapy, Lu-177

## Abstract

**Background:**

For dosimetry, the demand for whole-body SPECT/CT imaging, which require long acquisition durations with dual-head Anger cameras, is increasing. Here we evaluated sparsely acquired projections and assessed whether the addition of deep-learning-generated synthetic intermediate projections (SIPs) could improve the image quality while preserving dosimetric accuracy.

**Methods:**

This study included 16 patients treated with ^177^Lu-DOTATATE with SPECT/CT imaging (120 projections, 120P) at four time points. Deep neural networks (CUSIPs) were designed and trained to compile 90 SIPs from 30 acquired projections (30P). The 120P, 30P, and three different CUSIP sets (30P + 90 SIPs) were reconstructed using Monte Carlo-based OSEM reconstruction (yielding 120P_rec, 30P_rec, and CUSIP_recs). The noise levels were visually compared. Quantitative measures of normalised root mean square error, normalised mean absolute error, peak signal-to-noise ratio, and structural similarity were evaluated, and kidney and bone marrow absorbed doses were estimated for each reconstruction set.

**Results:**

The use of SIPs visually improved noise levels. All quantitative measures demonstrated high similarity between CUSIP sets and 120P. Linear regression showed nearly perfect concordance of the kidney and bone marrow absorbed doses for all reconstruction sets, compared to the doses of 120P_rec (R^2^ ≥ 0.97). Compared to 120P_rec, the mean relative difference in kidney absorbed dose, for all reconstruction sets, was within 3%. For bone marrow absorbed doses, there was a higher dissipation in relative differences, and CUSIP_recs outperformed 30P_rec in mean relative difference (within 4% compared to 9%). Kidney and bone marrow absorbed doses for 30P_rec were statistically significantly different from those of 120_rec, as opposed to the absorbed doses of the best performing CUSIP_rec, where no statistically significant difference was found.

**Conclusion:**

When performing SPECT/CT reconstruction, the use of SIPs can substantially reduce acquisition durations in SPECT/CT imaging, enabling acquisition of multiple fields of view of high image quality with satisfactory dosimetric accuracy.

**Supplementary Information:**

The online version contains supplementary material available at 10.1186/s40658-024-00655-x.

## Background

Single-photon emission computed tomography (SPECT) combined with computed tomography (CT) is an imaging modality within the nuclear medicine field. This system is used for diagnostic purposes, monitoring treatment effects and to enable dosimetry after molecular radiotherapy. Molecular radiotherapy includes Lutetium-177 (^177^Lu) labelled ligands that bind to somatostatin-receptors (SSR), well-established for patients with neuroendocrine tumors, or prostate-specific membrane antigen (PSMA), recently approved with promising results for patients with metastatic castration-resistant prostate cancer [1–4]. Dosimetry is important in the quest to achieve personalised therapies. According to the International Commission on Radiological Protection Publication 140 [5], dosimetry should be performed the same as is required for external beam radiotherapy. Dosimetry allows monitoring of absorbed doses during successive therapy cycles, to avoid radiation-related injuries of organs at risk and to establish absorbed doses to tumors, and might help to optimise treatments and improve patient outcome. In a review article from 2014, Strigari et al. [6] found strong indications that personalised treatments would improve outcome and increase survival. Personalised dosimetry has also shown statistically significantly improved results compared to standard dosimetry for selective internal radiation therapy of advanced hepatocellular carcinoma with yttrium-90 microspheres [7]. Furthermore, research suggests that dosimetry might be used in pre-therapy imaging, enabling more accurate decision-making about whether a patient will benefit from molecular radiotherapy [8, 9]. Altogether, dosimetry shows great promise and needs to be further implemented [10], which will consequently increase the need for SPECT/CT imaging.

Recommendations from the European Association of Nuclear Medicine (EANM) state that the dosimetry imaging protocol should include at least three imaging time points, well separated in time [11]. The acquisition duration with conventional dual-head Anger cameras is a limiting factor and whole-body SPECTs with low noise levels are not easily obtained, especially for late time points after administration. The EANM guidelines recommend using 30–40 s frames and 60–120 projections for imaging after ^177^Lu-SSR therapy [11]. This leads to an acquisition time of 15–40 min for one field of view (FOV) covering approximately 40 cm of the body, with subsequent CT. In ^177^Lu-PSMA therapy, the organs at risk include the kidneys and bone marrow, well-documented risk organs in ^177^Lu-SSR therapy [12], and the salivary glands [13, 14]. Thus, imaging of all risk organs would require multiple FOVs or whole-body SPECT. The acquisition duration per FOV could be decreased by acquiring fewer (i.e. sparsely acquired) projections; however, this would reduce image quality. The noise level in ^177^Lu SPECT imaging is high, especially in imaging at late time points. Several centres have reduced acquisition durations to implement kidney dosimetry in a busy clinical reality, with limited numbers of available SPECT cameras. However, this might reduce the accuracy not only in CT based kidney dosimetry, but especially the accuracy for CT based bone marrow dosimetry due to increased image noise from the low uptake in the radiosensitive bone marrow [15], Nevertheless, to our knowledge, the impact of reduced acquisition durations on kidney and bone marrow dosimetry has not been fairly evaluated.

Here we aimed to evaluate the accuracy of kidney and bone marrow absorbed doses for SPECT images reconstructed using 30 projections (30P) instead of 120 projections (120P). Additionally, we analysed how synthetic intermediate projections (SIPs) generated by a deep neural network impacted image quality as well as kidney and bone marrow dosimetry. The deep neural network was designed and trained to generate 90 SIPs from an input of 30P. This network has previously been shown to perform well in reconstructions of ^177^Lu-DOTATATE and Indium-111 (^111^In)-octreotide images, showing high structural similarity between acquired projections and SIPs, and improved image quality in reconstructed images compared to reconstruction of 30P [16, 17]. However, this study only evaluated images from one day post administration and did not evaluate any dosimetry. In this study, the network has been retrained with an expansion of the training data to see if further improvements could be accomplished. The evaluation has been extended to include imaging of different noise levels (time points) as well as kidney and bone marrow dosimetry.

## Methods

### The convolutional neural network

The utilised convolutional neural network was the deep Convolutional U-net-shaped neural network for generation of Synthetic Intermediate Projections (CUSIP), presented by Rydén et al. in 2021 [16]. A schematic illustration of the network structure can be found in the supplementary material (Fig. S1). This network generates 90 SIPs from an input of 30P (down-sampled from the acquired 120P choosing every fourth projection starting with the first, i.e., projections 1,5,9…117). To generate SIPs the CUSIP was trained, using 120P as reference, three times to yield three different sets of SIPs: projections 2,6,10…118; projections 3,7,11…119, and projections 4,8,12…120. These 90 SIPs were added to the 30P input, forming a so-called CUSIP set of 120 projections.

For this study, the network was retrained, with expansion of the training material of ^177^Lu-DOTATATE images. Training parameters used were the same as Rydén et al. [16], only this time the network was trained for 300 epochs. All imaging time points were mixed during training (with 96% being one day post administration due to the historically used hybrid planar-SPECT/CT protocol). We compared two different loss functions, L1 – Least Absolute Deviations and L2 – Least Square Errors, used to minimize the error between the SIPs and the acquired projections during training. Further, we added ^111^In-octreotide images to the training material with L2 chosen as the loss function (as it outperformed L1 in the comparison, see the results section), forming the extended L2 network (Table 1).

**Table 1:** The networks trained in this study and the number (no.) of examinations in the training, validation, and test groups

Network	Loss function	Radiopharmaceuticals in training data	Training data (no.)	Validation data (no.)	Test data (no.)
1	L1	Lu-177-DOTATATE	486	10	64 (16 × 4)
2	L2	Lu-177-DOTATATE	486	10	64 (16 × 4)
3	L2	Lu-177-DOTATATE (23%) + In-111-octreotide (77%)	2140	10	64 (16 × 4)

The test group included 16 patients with four imaging time points each

### SPECT reconstruction

The 16 test patients, each with five sets of projection data (Table 2), were reconstructed using the Sahlgrenska Academy Reconstruction Code (SARec), an ordered subset expectation maximization (OSEM)-based reconstruction algorithm that uses Monte Carlo (MC) simulations in the forward projection in the iterative process to correct for photon attenuation, scattering (in the patient and the collimator), and resolution recovery [18]. Resolution recovery correction was also applied in the back projection. The OSEM reconstruction was performed with 10 iterations and 6 subsets using an in-house developed software program. MC simulations within the reconstruction were performed with 200 photons/voxel emitted in an angular range of 0.06 radians.

**Table 2:** SPECT reconstruction and projection sets, with corresponding terminology. Networks 1, 2 and 3 refer to the networks presented in Table 1

SPECT reconstruction set	Projection set
120P_rec	Original 120 projections (120P)
30P_rec	Every fourth projection of the 120P (30P)
CUSIP_rec_L1	30P + 90 SIPs generated by network 1 (CUSIP_L1)
CUSIP_rec_L2	30P + 90 SIPs generated by network 2 (CUSIP_L2)
CUSIP_rec_L2_ext_	30P + 90 SIPs generated by network 3 (CUSIP_L2_ext_)

### Subjects and image acquisition

For this study, we selected 2214 examinations in which SPECT/CT imaging was performed after treatment with ^177^Lu-DOTATATE or during examination with ^111^In-octreotide, at Sahlgrenska University Hospital, between 2003 and 2021. The inclusion criterium was SPECT/CT acquisition with 120P. The retrospective use of image data was approved by the Swedish Ethics Review Board, and the need for written informed consent was waived.

The examinations were performed using dual-head Anger cameras of models Millenium VG Hawkeye, Infinia Hawkeye 4, Discovery 670, and two Discovery 670 Pro, all from General Electric Medical Systems (Milwaukee, WI, USA). The crystal thickness was 3/8” for Infinia Hawkeye 4, and 5/8” for the other cameras. Acquisitions were performed with medium-energy general purpose (MEGP) parallel-hole collimators. The ^177^Lu-DOTATATE acquisitions were performed using the 208 keV photon peak of ^177^Lu, with an energy window of ± 10%. The ^111^In-octreotide acquisitions were performed using the 171 keV and 245 keV photon peaks, both with energy windows of ± 10%. These two energy windows were acquired and summed in the same image (not possible to separate in retrospect). Imaging was performed 1 day after administration of ^177^Lu-DOTATATE or ^111^In-octreotide. 4% of the ^177^Lu-DOTATATE acquisitions were from other time points than day 1 (range from day 0 to 7). The acquisition duration was 30 s/frame and 120P in step-and-shoot mode. The matrix size was 128 × 128, with a pixel size and slice thickness of 4.42 mm. For the CT, the matrix size was 256 × 256 for the Infinia Hawkeye 4, and 512 × 512 for the other cameras. The slice thickness was 5 mm for all CT cameras. The pixel size was 2.21 mm for the Infinia camera, 1.10 mm for the Millenium camera, and 0.98 mm for the Discovery 670 and Discovery 670 Pro cameras. All imaging acquisition parameters are presented in Table 3.

**Table 3:** Imaging acquisition parameters for the cameras used for the training, validation and test data

	Millenium VG Hawkeye	Infinia Hawkeye 4	Discovery 670	Discovery 670 Pro
Crystal thickness	5/8”	3/8”	5/8”	5/8”
Collimator	MEGP	MEGP	MEGP	MEGP
Energy window Lu-177	208 keV ± 10%	208 keV ± 10%	208 keV ± 10%	208 keV ± 10%
Energy window In-111*	171 keV ± 10% 245 keV ± 10%	171 keV ± 10% 245 keV ± 10%	171 keV ± 10% 245 keV ± 10%	171 keV ± 10% 245 keV ± 10%
Acquisition (step-and-shoot)	30 s/projection, 120 projections	30 s/projection, 120 projections	30 s/projection, 120 projections	30 s/projection, 120 projections
Matrix size SPECT	128 × 128	128 × 128	128 × 128	128 × 128
Matrix size CT	512 × 512	256 × 256	512 × 512	512 × 512
Pixel size & Slice thickness SPECT	4.42 mm	4.42 mm	4.42 mm	4.42 mm
Pixel size CT Slice thickness CT	1.10 mm 5 mm	2.21 mm 5 mm	0.98 mm 5 mm	0.98 mm 5 mm

* The two energy windows for In-111 were acquired in the same image

### Evaluation/test group

To evaluate the performance of the networks, a test group including 16 sequential patients, 8 men and 8 women, treated with ^177^Lu-DOTATATE between 2019 and 2021 was selected. The mean age was 70 years (range 46–86 years). The patients were treated with a mean activity ± standard deviation (SD) of 7601 ± 123 MBq of ^177^Lu-DOTATATE, and imaging was performed after administration at day 0 (D0, range: 1.3–5.8 h), day 1 (D1, range: 19.5–24.5 h), day 2 (D2, range: 43.8–51.2 h), and day 7 (D7, range: 168.0–173.0 h). Each imaging time point included SPECT/CT with 120P over the abdomen, acquired using the two Discovery 670 Pro cameras described above, yielding a total of 64 image sets in the test group. For the projections, the original 120P served as the ground truth, with which the projection sets from the CUSIPs were compared. Similarly for the reconstructed images, the reconstruction of 120P (120P_rec) served as the ground truth, with which the reconstructions of the CUSIP sets, as well as reconstructions of 30P (30P_rec), were compared. Table 2 defines the terminology of the projection and reconstruction sets.

### Quantitative measures

To evaluate the similarity between the acquired projections and the CUSIP sets, as well as 30P_rec and CUSIP_recs compared to 120P_rec, we used four quantitative measures: normalised root mean square error (NRMSE), normalised mean absolute error (NMAE), peak signal-to-noise ratio (PSNR), and structural similarity (SSIM). The root mean square error (RMSE) is the square root of the mean of quadratic differences between the image (IM) and the reference image (RI) and measures the average magnitude of the error in unit pixels (for projections) or voxels (for reconstructions). To allow comparisons between different acquisition time points with their different count levels, the RMSE was normalised to the mean pixel or voxel value of the RI (Eq. 1).

1 $$\text{NRMSE}=\frac{\sqrt{\frac{1}{\text{nml}}{\sum }_{x}^{n}\sum _{y}^{m}\sum _{z}^{l}{(\text{IM}\left(x,y,z\right)-\text{RI}\left(x,y,z\right))}^{2}}}{\stackrel{-}{\text{R}\text{I}}(x,y,z)}$$

n, m, and l are the number of voxels in each direction; and x, y, and z are the coordinates in the SPECT image. $\stackrel{-}{\text{R}\text{I}}$ is the mean voxel value in the reference image. For the projection images, IM and RI represent 2D images instead. The mean absolute error (MAE) is the average of the absolute differences between IM and RI (in pixels/voxels) and, like RMSE, is normalised to the mean pixel/voxel value of the RI (Eq. 2).

2 $$\text{NMAE}=\frac{\frac{1}{\text{nml}}{\sum }_{x}^{n}\sum _{y}^{m}\sum _{z}^{l}\left|\text{I}\text{M}\left(x,y,z\right)-\text{R}\text{I}\left(x,y,z\right)\right|}{\stackrel{-}{\text{R}\text{I}}(x,y,z)}$$

The NRMSE and NMAE are measures of the magnitude of the error in relation to the mean pixel/voxel value, and lower values imply lower difference. The PSNR (in decibels), can be used to compare the image quality between IM and RI, and is derived from the RMSE (Eq. 3):

3 $$\text{PSNR}=20{\text{log}}_{10}\left(\frac{MAX}{\text{RMSE}}\right)$$

MAX is the maximum pixel/voxel value in any of the images. The PSNR describes the maximum possible pixel/voxel value in relation to the noise (in terms of the introduced error, RMSE), and could be considered a measure of contrast. Higher PSNR indicates a better match between the IM and RI in terms of image quality. NRMSE, NMAE and PSNR rely on numeric comparisons, and does not reflect the human visual system. To appreciate the perceived image quality, SSIM assesses perceptual image quality by considering image degradation as perceived change in structural information (Eq. 4). SSIM ranges from 0 to 1, with a higher value implying a higher similarity between the images.

4 $$\text{SSIM}\left(\text{IM,RI}\right)=\frac{(2{\mu }_{\text{IM}}{\mu }_{\text{RI}}+{c}_{1})(2{\sigma }_{\text{IMRI}}+{c}_{2})}{({{\mu }_{\text{IM}}}^{2}{{\mu }_{\text{RI}}}^{2}+{c}_{1})(2{\sigma }_{\text{IM}}{\sigma }_{\text{RI}}+{c}_{2})}$$

µ is the average voxel value, σ^2^ is the variance, and σ_IMRI_ is the covariance of IM and RI. Additionally, c_1_ and c_2_ are variables used to stabilize the division and depend on the dynamic range of the pixel or voxel values [19].

### Dosimetry

Kidney dosimetry was performed for the 16 patients in the test group, and for all reconstruction sets. Bone marrow dosimetry was performed for 15 patients, among whom 6 patients had confirmed bone metastases. 1 patient with severe bone metastases involvement was excluded due to the strong influence of uptake in the metastases on the nearby bone marrow cavities. The dosimetry was based on reconstructed SPECT images at days 0, 1, 2, and 7 post-administration, and biexponential curve fits were used for the kinetics of the activity concentrations in segmented volumes of interest (VOIs). The kidney VOIs were manually delineated on CT images and for the bone marrow, 4 ml spherical VOIs were used [20, 21], to mitigate the impact of the partial volume effect. The sphere VOIs were placed in the CT images inside the vertebras T9 – L5 (the interval included in the FOV) and were manually modified in a few cases to avoid bone metastases or calcifications. Hemmingsson et al. has shown that the red marrow has a specific uptake of Lu-177-DOTATATE, hence, a volume fraction of 0.57 (mean of men and women, and of lumbar- and thoracic vertebras) was used to scale the activity concentration in the vertebra VOIs (which also contain yellow marrow and trabecular bone) [15]. The VOIs for each time point were used for all reconstruction sets. Calibration factors for each camera were used. For kidney dosimetry, specific recovery coefficients (RCs) for each reconstruction method were estimated to correct the kidney activity concentration for partial volume effect. The RCs were calculated using MC simulations of raw data (120P) from a typical kidney VOI. The VOI was retrieved from a patient and filled with a uniform, known activity and the MC simulations were executed using the patient’s CT images. The data were down-sampled to 30P, and the CUSIPs were used to compile the three CUSIP projection sets. All five projection sets were reconstructed with SARec OSEM, and the activity within the kidney VOI for each reconstruction set was established and compared to the known activity. For the bone marrow the partial volume effect was disregarded as the sphere was placed in a homogenous surrounding. The time-integrated activity concentration was determined by integrating the curve-fitted bi-exponential function from time zero to infinity. When calculating the absorbed dose to kidneys, local energy deposition of the electrons was assumed and the dose contribution from photons was disregarded. The absorbed fraction for the red bone marrow was set to 0,65 (mean of men and women) [15]. All dosimetric calculations and respective figures were performed and produced with MATLAB version: 9.11.0 (R2021b) (The MathWorks Inc, Natick, Massachusetts, United States of America).

### Statistical analysis

Statistical analyses were performed using IBM SPSS Statistics version 29 (IBM Corporation, Armonk, New York, USA). For the quantitative measures, one-way ANOVA for repeated measures was performed with adjustment for multiple comparisons (Bonferroni). A *P* value of < 0.05 was considered to indicate statistical significance. For the kidney and bone marrow dosimetry, dependent samples t-tests were used with Bonferroni adjustment of the significance level (*P* value < 0.0125) for 4 consecutive tests.

## Results

**Fig. 1:**
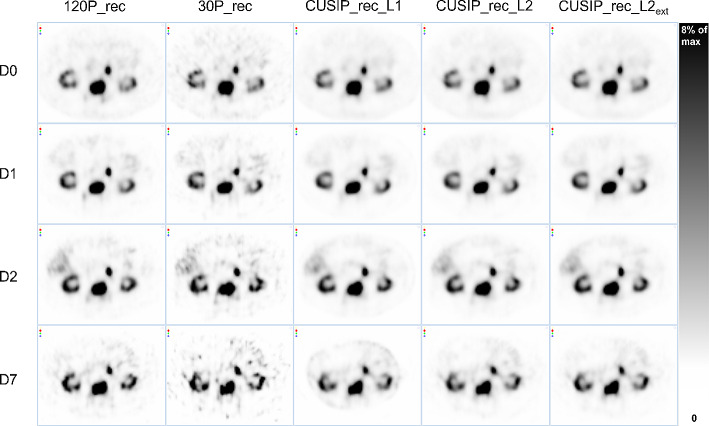
SPECT images of the abdomen for all reconstruction sets and imaging time points: day (D) 0, D1, D2, and D7 post-administration

Figure 1 presents a visual comparison of the five reconstruction sets and imaging time points (D0, D1, D2, and D7). The noise was evident in 30P_rec, especially for the last time point (D7). The reconstructions including SIPs, the CUSIP_recs, were smooth in their appearance, compared to 30P_rec and even 120P_rec. Line profiles corresponding to the images in Fig. 1 can be found in the supplementary material (Figs. S2, S3).

The quantitative measures, presented as the mean values from the 16 patients in the test group, showed good agreement between the CUSIP sets and the original projections (Table 4) and between the reconstructions of 30P and the CUSIP sets compared to the reconstruction of the original raw data (Table 5). The values of NRMSE and NMAE were low overall, with a slight increase at later time points. PSNR and SSIM also deteriorated at later time points. The structural similarities for the reconstructions were close to 1, implying nearly perfect similarity. Compared to CUSIP_L1, CUSIP_L2 performed better overall (both for projections and reconstructions, and for all days), especially in D7 images. Therefore, L2 was used as the loss function in the extended training material (CUSIP_L2_ext_). Furthermore, CUSIP_L2_ext_ resulted in improved values of all measures and all days (16 combinations; 4 measures x 4 days) compared to the other methods, for both projections (Table 4) and reconstructions (Table 5). There were statistically significant differences between CUSIP_L2_ext_ and CUSIP_L1 for projections: all 16 combinations and for reconstructions: 14 of 16 combinations. Between CUSIP_L2_ext_ and CUSIP_L2 there were statistically significant differences for projections: all combinations and reconstructions: 14 of 16 combinations. Compared to 30P_rec, CUSIP_rec_L2_ext_ was significantly improved, statistically, for all 16 combinations (Table 5).

**Table 4:** Quantitative measures showing the agreement between the CUSIP sets and the original projections (120P), for day (D) 0, 1, 2, and 7

Measure	Projection Set	D0	D1	D2	D7
NRMSE	*CUSIP_L1*	0.42	0.58	0.68	1.21
	*CUSIP_L2*	0.41	0.57	0.67	1.18
	*CUSIP_L2*_*ext*_	**0.35**	**0.49**	**0.58**	**1.01**
NMAE	*CUSIP_L1*	0.19	0.25	0.30	0.51
	*CUSIP_L2*	0.20	0.26	0.31	0.57
	*CUSIP_L2*_*ext*_	**0.15**	**0.20**	**0.23**	**0.42**
PSNR	*CUSIP_L1*	39.5	35.5	35.2	33.0
	*CUSIP_L2*	39.6	35.7	35.3	33.2
	*CUSIP_L2*_*ext*_	**40.9**	**37.1**	**36.7**	**34.7**
SSIM	*CUSIP_L1*	0.91	0.88	0.86	0.77
	*CUSIP_L2*	0.91	0.89	0.87	0.79
	*CUSIP_L2*_*ext*_	**0.94**	**0.92**	**0.91**	**0.85**

Error is indicated by NRMSE and NMAE, with lower error implying better agreement. Image quality is indicated by PSNR and SSIM, with higher values implying better agreement. Best results for each measure and day in bold

**Table 5:** Quantitative measures showing the agreement between the reconstruction sets (30P_rec and the CUSIP_recs) and the reconstruction of the original projections (120_rec) for day (D) 0, 1, 2, and 7

Measure	Reconstruction Set	D0	D1	D2	D7
NRMSE	*30P_rec*	0.734	1.066	1.254	2.260
	*CUSIP_rec _L1*	0.642	0.864*	1.001*	1.766*
	*CUSIP_rec _L2*	0.615*	0.863*	1.002*	1.622*
	*CUSIP_rec _L2*_*ext*_	0.587*	0.828*	0.960*	1.581*
NMAE	*30P_rec*	0.162	0.206	0.239	0.414
	*CUSIP_rec _L1*	0.119*	0.149*	0.174*	0.329*
	*CUSIP_rec _L2*	0.116*	0.147*	0.170*	0.285*
	*CUSIP_rec _L2*_*ext*_	0.113*	0.143*	0.166*	0.278*
PSNR	*30P_rec*	52.8	50.7	50.3	49.5
	*CUSIP_rec _L1*	53.7	52.0*	51.5*	50.1
	*CUSIP_rec _L2*	54.0*	52.0*	51.5*	50.7
	*CUSIP_rec _L2*_*ext*_	54.3*	52.4*	51.8*	51.0*
SSIM	*30P_rec*	0.996	0.994	0.992	0.988
	*CUSIP_rec _L1*	0.9978*	0.9965*	0.995*	0.986
	*CUSIP_rec _L2*	0.9978*	0.9967*	0.9958*	0.992*
	*CUSIP_rec _L2*_*ext*_	0.9979*	0.9968*	0.9960*	0.993*

*Indicating a statistically significant difference compared to 30P_rec. Error is indicated by NRMSE and NMAE, with lower error implying better agreement. Image quality is indicated by PSNR and SSIM, with higher values implying better agreement. Best results for each measure and day in bold

**Fig. 2:**
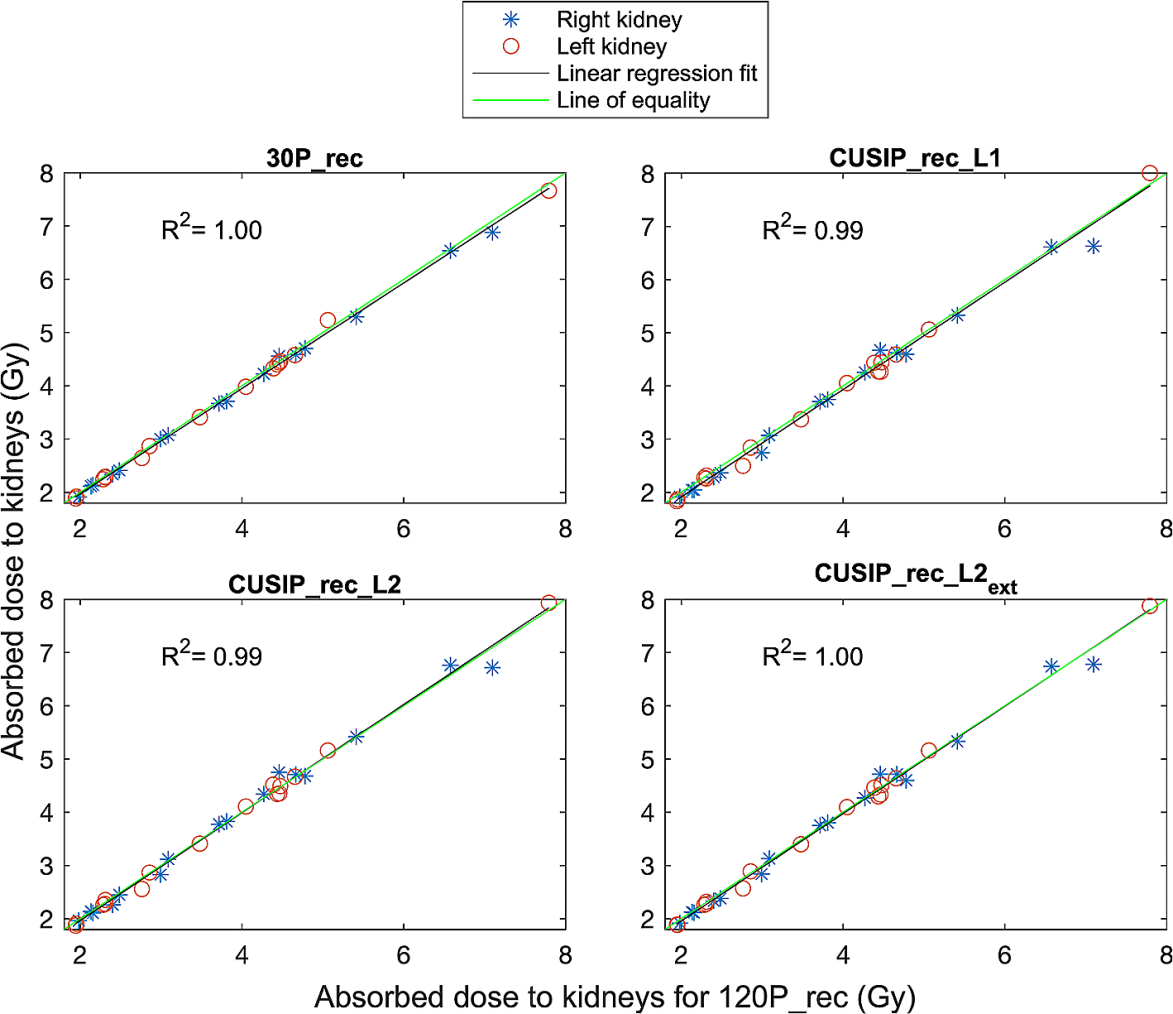
The dose relationship between the kidney absorbed doses (blue stars: right kidney, red circles: left kidney) calculated for each reconstruction set compared to the kidney absorbed doses calculated for 120P_rec and linear regression fits with corresponding R^2^ values

**Fig. 3:**
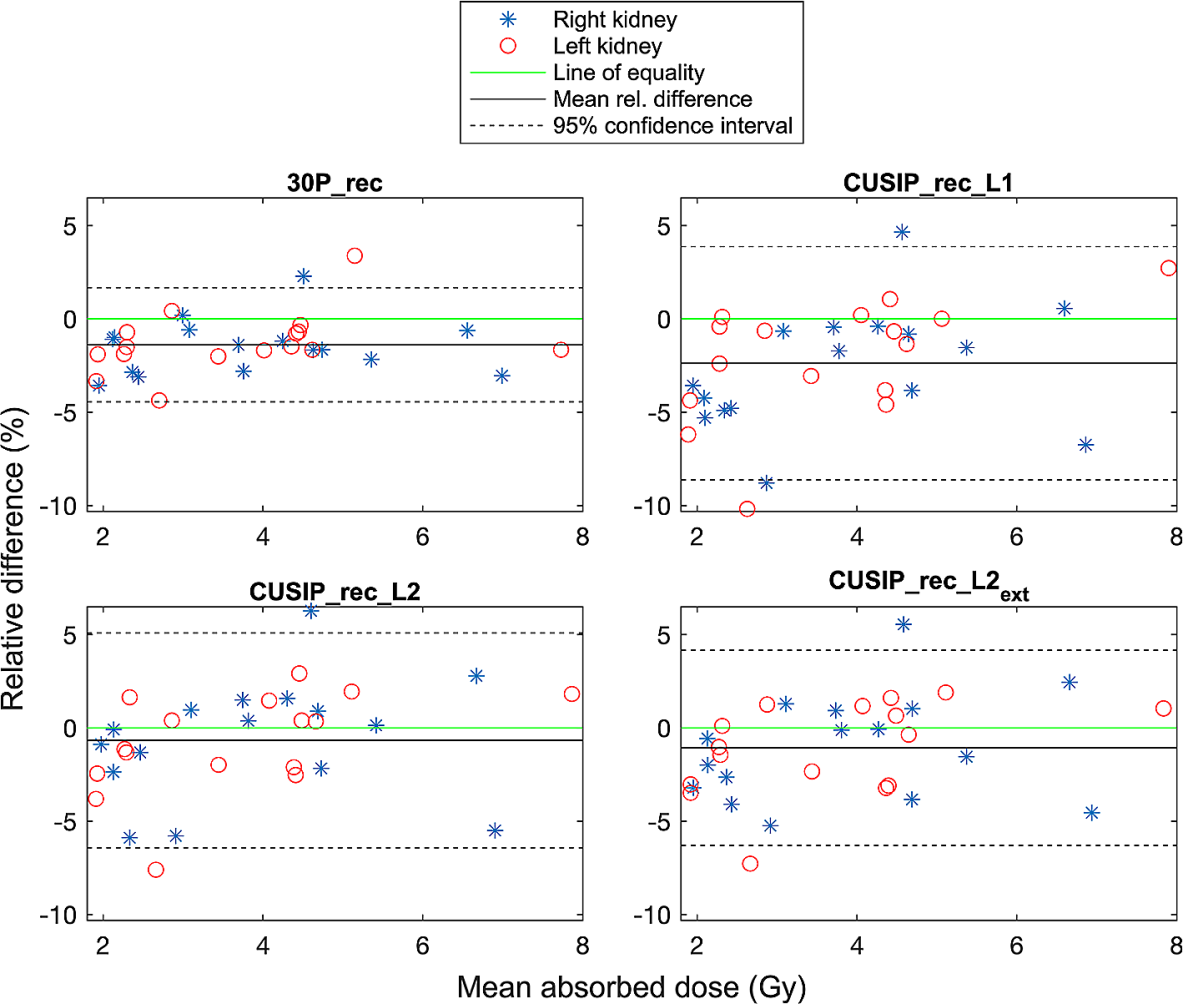
Bland-Altman plots showing the relative differences of the kidney absorbed doses for the 16 patients (right and left kidney shown separately) and for each reconstruction set compared to the mean absorbed doses of each method and 120P_rec, including mean relative difference and 95% confidence interval

The RC for each reconstruction method ranged from 0.81 to 0.87 (0.87 for 120P_rec and 30P_rec, 0.83 for CUSIP_rec_L1 and CUSIP_rec_L2_ext_ and 0.81 for CUSPI_rec_L2). Kidney dosimetry showed a nearly perfect concordance between the doses calculated from the different reconstruction sets and the doses calculated from 120P_rec (Fig. 2). Bland-Altman plots show the relative differences of the kidney absorbed doses compared to the mean of each method and 120P_rec (Fig. 3). All reconstruction sets exhibited a relative difference among the 16 patients of < 10% (the majority < 5%), with 30P_rec showing the narrowest confidence interval (CI). Table 6 presents the mean absorbed doses (left and right kidney, 16 patients), mean relative differences, and SDs of the relative differences (compared to the doses of 120P) for all reconstruction sets. The mean absorbed doses of 30P_rec and CUSIP_rec_L1 were significantly different from the mean dose of the reference, 120P_rec (Table 6).

**Table 6:** Mean kidney absorbed dose for 16 patients, left and right kidney, for all reconstruction sets

	Mean dose (Gy)	Mean rel. difference (%)	SD (%)	*P* value (95% CI)
120P_rec	3.79			
30P_rec	3.74*	−1.36	1.55	< 0.001 (− 0.071 to − 0.023)
CUSIP_rec_L1	3.72*	−2.30	3.10	0.004 (− 0.115 to − 0.024)
CUSIP_rec_L2	3.78	−0.62	2.91	0.639 (− 0.054 to 0.034)
CUSIP_rec_L2_**ext**_	3.76	−1.02	2.64	0.179 (− 0.066 to 0.013)

Mean relative difference and standard deviation (SD) of the relative differences compared to 120P_rec. *Indicating a statistically significant difference compared to 120P_rec

**Fig. 4:**
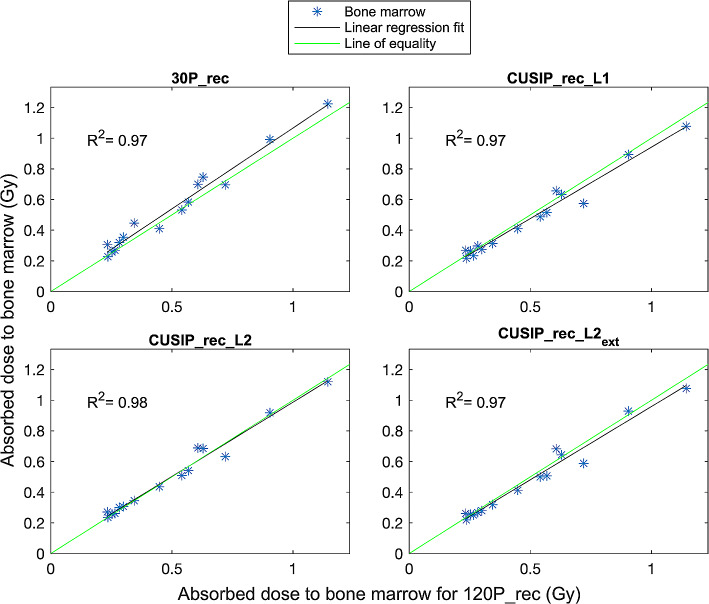
The dose relationship between the bone marrow absorbed doses calculated for each reconstruction set compared to those calculated for 120P_rec and linear regression fits with corresponding R^2^ values

**Fig. 5:**
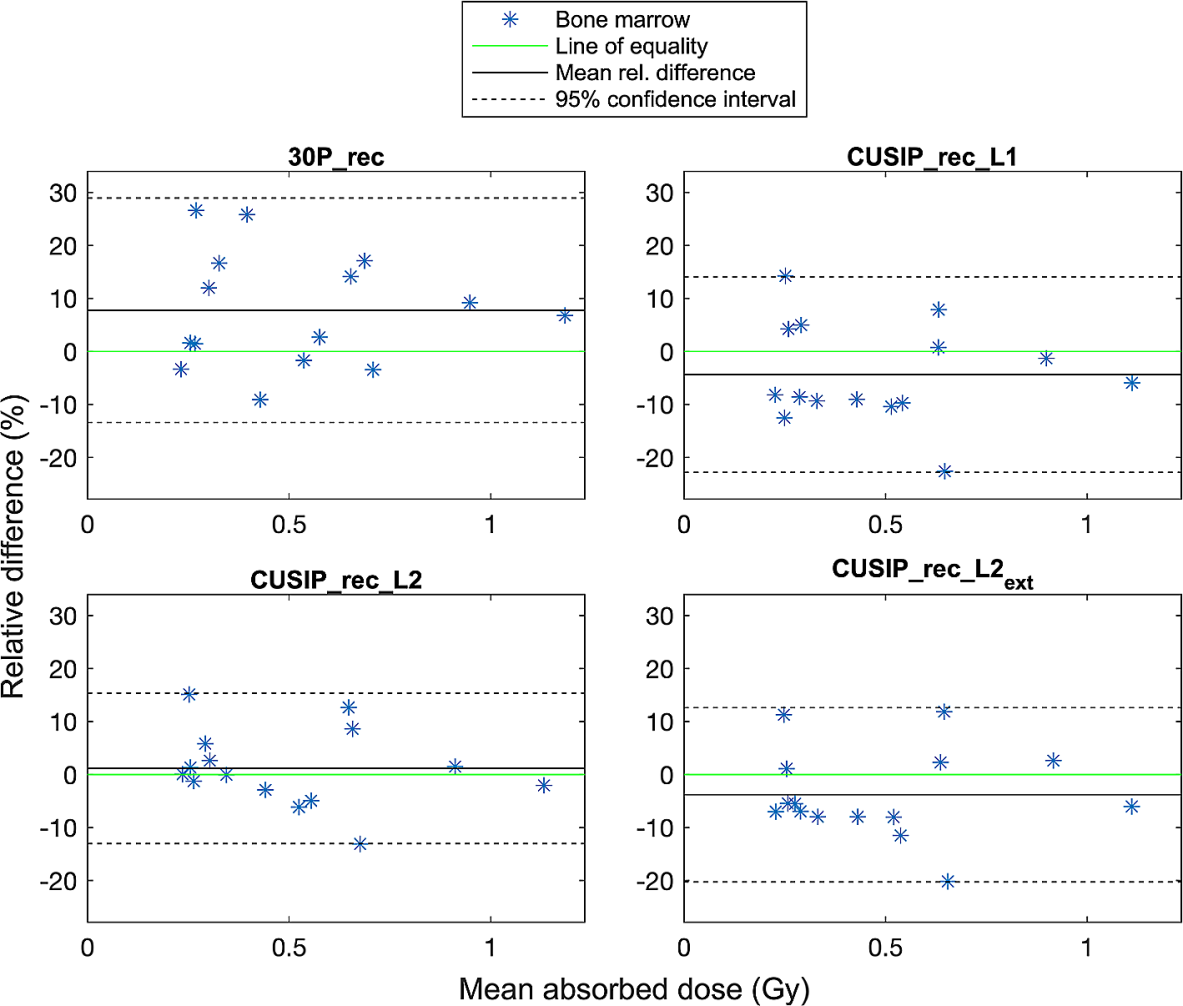
Bland-Altman plots showing the relative differences of the bone marrow absorbed doses for the 15 patients and for each reconstruction set compared to the mean absorbed doses of each method and 120P_rec, including mean relative difference and 95% confidence interval

Bone marrow dosimetry showed overall good agreement between the absorbed doses from 30P_rec and CUSIP_recs compared to 120P_rec (Fig. 4). There is, however, a wider spread in relative differences for bone marrow absorbed doses (Fig. 5) compared to kidney absorbed doses, especially for 30P_rec. 30P_rec overestimates the absorbed doses to bone marrow with nearly 10% (mean relative difference) compared to 120P_rec. The bone marrow mean absorbed doses, mean relative differences, and SDs are presented in Table 7. The doses of 30P_rec are, unlike the doses of CUSIP_recs, statistically significantly different from 120P_rec.

**Table 7:** Mean bone marrow absorbed dose for the 15 patients for all reconstruction sets

	Mean dose (Gy)	Mean rel. difference (%)	SD (%)	*P* value (95% CI)
120P_rec	0.498			
30P_rec	0.537*	8.70	11.98	0.010 (0.011 to 0.067)
CUSIP_rec_L1	0.475	−3.88	9.17	0.072 (− 0.050 to 0.002)
CUSIP_rec_L2	0.501	1.42	7.41	0.840 (− 0.020 to 0.024)
CUSIP_rec_L2_**ext**_	0.480	−3.44	8.24	0.153 (− 0.046 to 0.008)

Mean relative difference and standard deviation (SD) of the relative differences compared to 120P_rec. *Indicating a statistically significant difference compared to 120P_rec

## Discussion

In our present study, ^111^In-octreotide images were included as a strategy to increase the training material for increased accuracy of CUSIP. This strategy was successful, as it demonstrated consistently improved evaluation parameters throughout, and for all days, compared to the network trained using only ^177^Lu-DOTATATE images. The improvement was shown to be statistically significant for almost all combinations, but it is also important to acknowledge that the difference might not be clinically relevant. A smaller size of the training data set (CUSIP_L1/CUSIP_rec_L1 and CUSIP_L2/CUSIP_rec_L2) might be sufficient as the quantitative measures are very close to those of CUSIP_L2_ext_/ CUSIP_rec_L2_ext_ and the absorbed doses for CUSIP_rec_L2 are not significantly different, statistically, from those for 120P_rec. However, the absorbed doses for CUSIP_rec_L1 are significantly different, statistically, indicating that L2 is a more suitable loss function in this case. Although, important to remember is that the absence of significance does not mean they are equal.

The generalizability of CUSIP can be discussed. As of now, the training has been conducted on Lu-177 and In-111 which have similar photon energies and using only GE cameras with MEGP collimators. We expect SIPs to work well for all Lu-177 or In-111 ligands or any other radionuclide with photon energy in the same range. Using SIPs for technetium-99 m, or for different camera manufacturers or collimators, would have to be evaluated. Either including these variances into the training data or train different networks.

For patients treated with ^177^Lu-DOTATATE, a common imaging protocol is hybrid planar-SPECT/CT, in which the shape of the time-activity curve is determined by planar imaging at several time points, and SPECT/CT is acquired at one time point to establish the amplitude of the time-activity curve. This hybrid protocol has historically been used at Sahlgrenska University Hospital, with SPECT/CT acquired one day after administration of ^177^Lu-DOTATATE. Consequently, the ^177^Lu-DOTATATE training material in our trained networks comprised a majority (96%) of SPECTs from D1. Moreover, ^111^In-octreotide was also acquired one day post-injection, and the activity concentration level of ^111^In-octreotide D1 was in the range of the activity concentration level of ^177^Lu-DOTATATE D2, when comparing the means between 20 patients from the respective groups. Therefore, a majority of the training material, 77%, was ^111^In-octreotide (with activity concentration level of ^177^Lu-DOTATATE D2), 22% was ^177^Lu-DOTATATE D1; and 1% was ^177^Lu-DOTATATE from other days.

The quantitative measures NRMSE, NMAE, and PSNR indicated high similarity between the CUSIP sets and 120P, and between the reconstructions of 30P and CUSIP sets compared to 120P_rec. One drawback of these measures is that the absolute values are somewhat difficult to interpret since a large portion of the image is background (pixel/voxel values close to zero). However, our approach was in accordance with how others have done for similar comparisons. Notably, using cropped images would potentially exclude artefacts introduced by the network outside of the selected section, thus potentially rendering fallacious measures. Nevertheless, the NRMSE and NMAE values were very low with NRMSE around 0–1 pixel/voxel for the projections and 0–2 pixel/voxel for the reconstructions, and NMAE consistently under 1 pixel/voxel for both projections and reconstructions. According to all measures, the best performing network was the network trained with loss function L2 and the extended training material of both ^111^In and ^177^Lu images. CUSIP_rec_L2_ext_ was significantly different from 30P_rec in all measures and for all days.

The structural similarities between the reconstructions of the CUSIP sets and 120P_rec were close to 1, which is nearly perfect. However, the structural similarities were somewhat lower for the projection sets, probably because the projections covered the image matrix to a higher degree due to the PSF effect and, therefore, contained less background (pixel values close to zero) compared to the reconstructions.

The recovery coefficients retrieved showed that the different methods present slightly different partial volume effects. Therefore, it was valuable to include in the dosimetry calculations. The absorbed doses of the kidneys exhibited nearly perfect concordance between all reconstruction sets and 120_rec (Fig. 2). The 95% CI were narrow for all reconstruction sets with a majority of the absorbed doses within ± 5% (relative differences) from those for 120P_rec. However, statistically significant differences were found between the absorbed doses from 30P_rec and CUSIP_rec_L1 compared to 120P_rec (Table 6). A mean relative difference of under 3% and a narrow 95% CI for 30P_rec will, again, raise the question of what difference is clinically relevant. Regarding the bone marrow dosimetry, similar results were obtained with a high concordance between the absorbed doses for each reconstruction set compared to those for 120P_rec (Fig. 4). However, for bone marrow dosimetry, with higher noise in the VOIs, the relative differences compared to 120P_rec were higher than for the kidney absorbed doses (Fig. 5), especially for 30P_rec with relative differences of up to almost 30%. The bone marrow absorbed doses from 30P_rec were, unlike those from CUSIP_recs, significantly different, statistically, from 120P_rec, with a mean relative difference of almost 9%.

Altogether, even if kidney dosimetry based on sparsely acquired projections with 30P was sufficient, the visual image quality of 30P_rec is inadequate and was clearly improved by deep-learning generated synthetic projections, especially for late time points. The visual noise was improved not only compared to 30P_rec but also compared to 120P_rec. Another possible difficulty of using only 30P would be the manual VOI adjustment to the SPECT image in cases of organ movements, which would be considerably more difficult in noisy images, especially for late time points (as seen in Fig. 1). Rydén et al. also showed, in a visual comparison with an experienced nuclear medicine physician, that 30P_rec had unacceptable image quality with too much noise for clinical interpretation, whereas adding SIPs to the sparsely acquired projections generated reconstructed images with quality very similar to those of 120P_rec [16]. Also, considering bone marrow dosimetry, sparsely acquired projections with 30P is questionable due to the large mean relative difference in absorbed doses compared to those for 120P_rec and the large 95% CI with relative differences of up to 30%. Consequently, it appears that SPECT reconstructions with deep-learning-generated SIPs are superior to acquisitions with sparsely acquired projections, as SIPs improve the visual image quality and make kidney and bone marrow dosimetry feasible.

The use of SIPs seems to be a promising noise reduction technique, as it has previously yielded improved results compared to regular post filtering with Butterworth or Gaussian filters [16, 17]. Future studies should be conducted with the aim of examining the detectability of small lesions in SPECT/CT imaging using SIPs instead of standard filtering. Furthermore, it is important to mention the recent developments in SPECT imaging through new camera designs that enable 360-degree SPECT acquisitions. These cameras can accomplish whole-body SPECTs within a short time frame, due to improvements in sensitivity compared to conventional dual-head cameras [22]. Both the introduction of deep-learning methods, as CUSIP, and new camera designs are essential for enabling clinical whole-body SPECT and increasing patient comfort by reducing acquisition times.

## Conclusions

This study has demonstrated that SPECT/CT reconstruction using SIPs generated by a deep neural network is feasible, showing good agreement with reconstruction of 120P. Image quality was significantly improved compared to reconstructions of 30P. Additionally, the noise was visually improved compared to 30P_rec and 120P_rec. Reconstructions based on SIPs are also feasible for kidney and bone marrow dosimetry, outperforming sparsely acquired projections (30P_rec) for especially bone marrow dosimetry, where 30P_rec showed large deviations, statistically significant, from 120P_rec. This method can substantially reduce the image acquisition duration, thereby enabling the acquisition of multiple FOVs with clinically reasonable acquisition times.

### Electronic supplementary material

Below is the link to the electronic supplementary material.

Supplementary Material 1

## Data Availability

The data in the current study are available from the corresponding author upon reasonable request.

## Abbreviations

_rec: reconstruction of …
120P: 120 projections
30P: 30 projections
CI: Confidence interval
CT: Computed tomography
CUSIP: Convolutional U-net-shaped neural network for generation of Synthetic Intermediate Projections
D0, D1, …: Day 0, day 1, …
EANM: European Association of Nuclear Medicine
FOV: Field of view
IM: Image
MAE: Mean absolute error
MEGP: Medium-energy general purpose
NMAE: Normalized mean absolute error
NRMSE: Normalized root mean square error
OSEM: Ordered subset expectation maximization
PSMA: Prostate-specific membrane antigen
PSNR: Peak signal-to-noise ratio
RI: Reference image
RMSE: Root mean square error
SARec: Sahlgrenska Academy Reconstruction Code
SD: Standard deviation
SIPs: Synthetic intermediate projections
SPECT: Single-photon emission computed tomography
SSIM: Structural similarity
SSR: Somatostatin-receptor
VOI: Volume of interest

## References

[1] Gafita A, Rauscher I, Retz M, Knorr K, Heck M, Wester HJ (2019). Early experience of rechallenge (177)Lu-PSMA Radioligand Therapy after an initial good response in patients with advanced prostate Cancer. J Nucl Med.

[2] Gafita A, Heck MM, Rauscher I, Tauber R, Cala L, Franz C (2020). Early prostate-specific Antigen Changes and Clinical Outcome after (177)Lu-PSMA Radionuclide treatment in patients with metastatic castration-resistant prostate Cancer. J Nucl Med.

[3] Rahbar K, Ahmadzadehfar H, Kratochwil C, Haberkorn U, Schäfers M, Essler M (2017). German Multicenter Study investigating 177Lu-PSMA-617 Radioligand therapy in advanced prostate Cancer patients. J Nucl Med.

[4] Sartor O, de Bono J, Chi KN, Fizazi K, Herrmann K, Rahbar K (2021). Lutetium-177-PSMA-617 for metastatic castration-resistant prostate Cancer. N Engl J Med.

[5] Yonekura Y, Mattsson S, Flux G, Bolch WE, Dauer LT, Fisher DR (2019). ICRP publication 140: Radiological Protection in Therapy with Radiopharmaceuticals. Ann ICRP.

[6] Strigari L, Konijnenberg M, Chiesa C, Bardies M, Du Y, Gleisner KS (2014). The evidence base for the use of internal dosimetry in the clinical practice of molecular radiotherapy. Eur J Nucl Med Mol Imaging.

[7] Garin E, Tselikas L, Guiu B, Chalaye J, Edeline J, de Baere T (2021). Personalised versus standard dosimetry approach of selective internal radiation therapy in patients with locally advanced hepatocellular carcinoma (DOSISPHERE-01): a randomised, multicentre, open-label phase 2 trial. Lancet Gastroenterol Hepatol.

[8] Müller C, Fischer E, Behe M, Köster U, Dorrer H, Reber J (2014). Future prospects for SPECT imaging using the radiolanthanide terbium-155 - production and preclinical evaluation in tumor-bearing mice. Nucl Med Biol.

[9] Taprogge J, Abreu C, Yusuf S, Ainsworth G, Phillip RH, Gear JI (2023). The role of Pretherapy Quantitative Imaging and Dosimetry in Radioiodine Therapy for Advanced thyroid Cancer. J Nucl Med.

[10] Lawhn-Heath C, Hope TA, Martinez J, Fung EK, Shin J, Seo Y (2022). Dosimetry in radionuclide therapy: the clinical role of measuring radiation dose. Lancet Oncol.

[11] Sjögreen Gleisner K, Chouin N, Gabina PM, Cicone F, Gnesin S, Stokke C (2022). EANM dosimetry committee recommendations for dosimetry of 177Lu-labelled somatostatin-receptor- and PSMA-targeting ligands. Eur J Nucl Med Mol Imaging.

[12] Ljungberg M, Celler A, Konijnenberg MW, Eckerman KF, Dewaraja YK, Sjogreen-Gleisner K (2016). MIRD Pamphlet 26: Joint EANM/MIRD guidelines for quantitative 177Lu SPECT Applied for Dosimetry of Radiopharmaceutical Therapy. J Nucl Med.

[13] Heynickx N, Herrmann K, Vermeulen K, Baatout S, Aerts A. The salivary glands as a dose limiting organ of PSMA- targeted radionuclide therapy: a review of the lessons learnt so far. Nucl Med Biol. 2021;98–9. 10.1016/j.nucmedbio.2021.04.003. 10.1016/j.nucmedbio.2021.04.003 34020337

[14] Taïeb D, Foletti JM, Bardiès M, Rocchi P, Hicks RJ, Haberkorn U (2018). PSMA-Targeted Radionuclide Therapy and salivary gland toxicity: why does it Matter?. J Nucl Med.

[15] Hemmingsson J, Svensson J, van der Meulen NP, Müller C, Bernhardt P (2022). Active bone marrow S-values for the low-energy electron emitter terbium-161 compared to S-values for lutetium-177 and yttrium-90. EJNMMI Phys.

[16] Rydén T, Van Essen M, Marin I, Svensson J, Bernhardt P (2021). Deep-Learning Generation of Synthetic Intermediate Projections improves (177)Lu SPECT images reconstructed with sparsely acquired projections. J Nucl Med.

[17] Rydén T, Emma W, Van Essen M, Svensson J, Bernhardt P (2021). Improvements of 111In spect images reconstructed with sparsely acquired projections by Deep Learning Generated Synthetic projections. Radiat Prot Dosimetry.

[18] Ryden T, Heydorn Lagerlof J, Hemmingsson J, Marin I, Svensson J, Bath M (2018). Fast GPU-based Monte Carlo code for SPECT/CT reconstructions generates improved (177)Lu images. EJNMMI Phys.

[19] Wang Z, Bovik AC, Sheikh HR, Simoncelli EP (2004). Image quality assessment: from error visibility to structural similarity. IEEE Trans Image Process.

[20] Del Prete M, Buteau F-A, Beauregard J-M (2017). Personalized 177Lu-octreotate peptide receptor radionuclide therapy of neuroendocrine tumours: a simulation study. Eur J Nucl Med Mol Imaging.

[21] Del Prete M, Arsenault F, Saighi N, Zhao W, Buteau F-A, Celler A (2018). Accuracy and reproducibility of simplified QSPECT dosimetry for personalized 177Lu-octreotate PRRT. EJNMMI Phys.

[22] Desmonts C, Bouthiba MA, Enilorac B, Nganoa C, Agostini D, Aide N (2020). Evaluation of a new multipurpose whole-body CzT-based camera: comparison with a dual-head anger camera and first clinical images. EJNMMI Phys.

