# Supplementary material for: Improvements of 177Lu SPECT images from sparsely acquired projections by reconstruction with deep-learning-generated synthetic projections
Source: EJNMMI Phys. 2024 Jun 28;11:53. doi: 10.1186/s40658-024-00655-x (PMC11213840; doi:10.1186/s40658-024-00655-x)
Supplement: Supplementary file 1 — Supplementary Material 1 [file 40658_2024_655_MOESM1_ESM.docx]

# Supplement

# Improvements of 177Lu SPECT Images from Sparsely Acquired Projections by Reconstruction with Deep-Learning-Generated Synthetic Projections

Emma Wikberg*^1,4^, Martijn van Essen^3^, Tobias Rydén^4^, Johanna Svensson^5^, Peter Gjertsson^2,3^, Peter Bernhardt^1,4^.

^1^Department of Medical Radiation Sciences, Institute of Clinical Sciences, Sahlgrenska Academy, University of Gothenburg, Gothenburg, Sweden

^2^Department of Molecular and Clinical Medicine, Institute of Medicine, Sahlgrenska Academy, University of Gothenburg, Gothenburg, Sweden

^3^Department of Clinical Physiology, Sahlgrenska University Hospital, 413 45, Gothenburg, Sweden

^4^Medical Physics and Medical Bioengineering, Sahlgrenska University Hospital, 413 45, Gothenburg, Sweden

^5^Department of Oncology, Institute of Clinical Sciences, Sahlgrenska Academy, University of Gothenburg, Gothenburg, Sweden

*Corresponding author:

Emma Wikberg, Gula Stråket 2B plan 3, 413 45 Gothenburg, Sweden, telephone: +46709136581, emma.wikberg@vgregion.se, ORCID: 0000-0002-9441-1059

## Methods
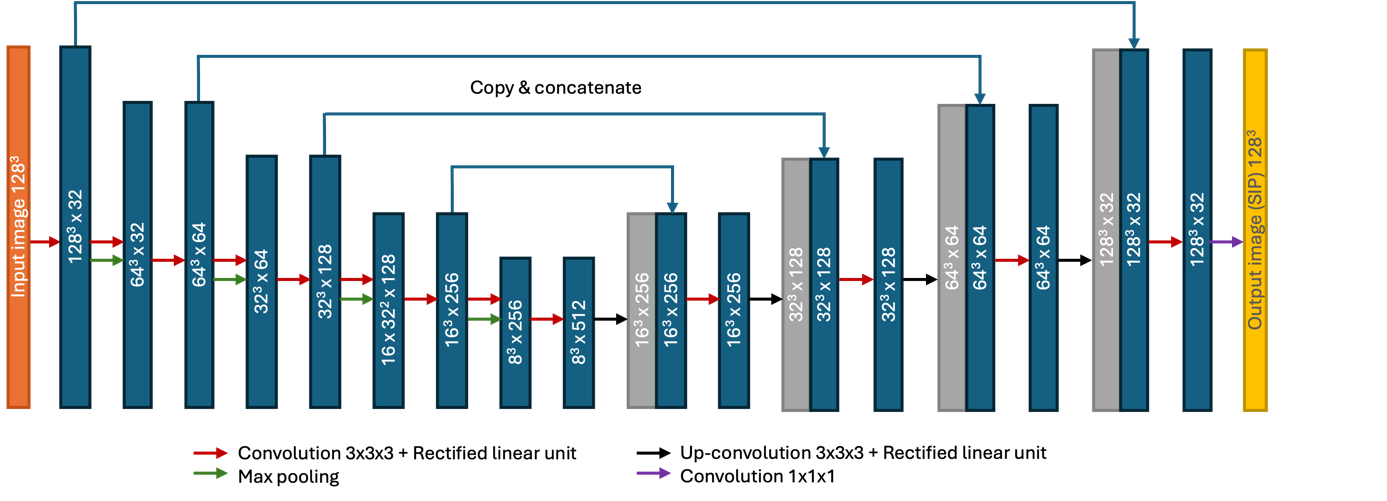


**Fig. S1** Schematic illustration of the network structure of CUSIP. Numbers are image size and number of features.

## Results


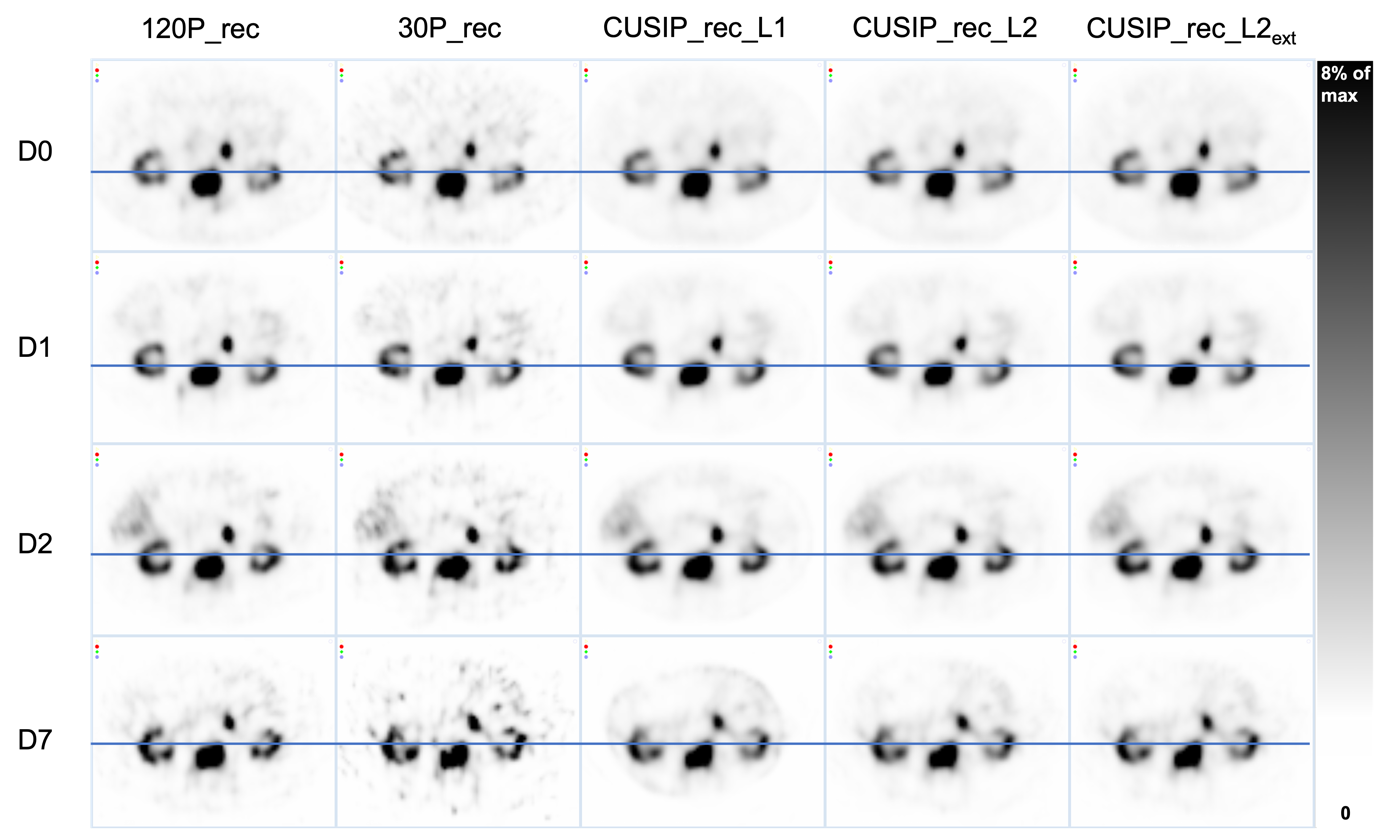


**Fig. S2** SPECT images of the abdomen for all reconstruction sets and imaging time points: day (D) 0, D1, D2, and D7 post-administration. Blue lines represent the positions of the line profiles shown in Fig. S3


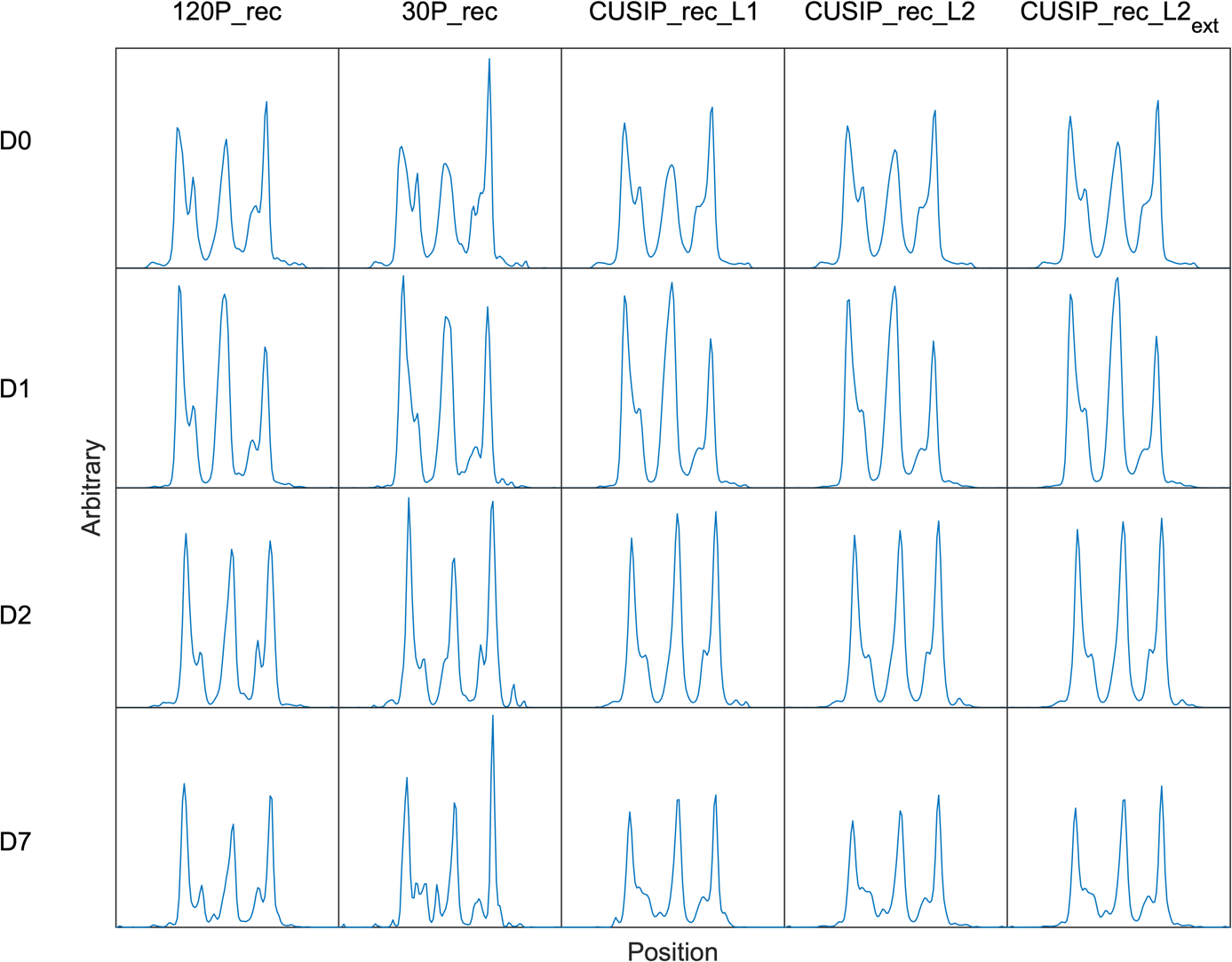


**Fig. S3** Corresponding line profiles for the SPECT images in Fig. S2. The line profiles go through the right kidney (first peak), a tumor (second peak), and left kidney (third peak). The y-axis is constant between the reconstruction sets for each day, respectively
